# Cross-Linked Hyaluronan Derivatives in the Delivery of Phycocyanin

**DOI:** 10.3390/gels10020091

**Published:** 2024-01-25

**Authors:** Francesca Terracina, Mario Saletti, Marco Paolino, Jacopo Venditti, Germano Giuliani, Claudia Bonechi, Mariano Licciardi, Andrea Cappelli

**Affiliations:** 1 Dipartimento di Scienze e Tecnologie Biologiche, Chimiche e Farmaceutiche (STEBICEF), Università degli Studi di Palermo, Via Archirafi 32, 90123 Palermo, Italy; francesca.terracina@unipa.it (F.T.); mariano.licciardi@unipa.it (M.L.); 2 Dipartimento di Biotecnologie, Chimica e Farmacia, Università degli Studi di Siena, Via Aldo Moro 2, 53100 Siena, Italy; mario.saletti@student.unisi.it (M.S.); paolino3@unisi.it (M.P.); jacopo.venditti@student.unisi.it (J.V.); giuliani5@unisi.it (G.G.); claudia.bonechi@unisi.it (C.B.)

**Keywords:** hyaluronic acid, ferulic acid, click-chemistry, crosslinking, hydrogel, drug delivery, phycocyanin

## Abstract

An easy and viable crosslinking technology, based on the “click-chemistry” reaction copper(I)-catalyzed azide-alkyne 1,3-dipolar cycloaddition (click-crosslinking), was applied to graft copolymers of medium molecular weight (i.e., 270 kDa) hyaluronic acid (**HA**) grafted with ferulic acid (**FA**) residues bearing clickable propargyl groups, as well as caffeic acid derivatives bearing azido-terminated oligo(ethylene glycol) side chains. The obtained crosslinked materials were characterized from the point of view of their structure and aggregation liability to form hydrogels in a water environment. The most promising materials showed interesting loading capability regarding the antioxidant agent phycocyanin (PC). Two novel materials complexes (namely **HA(270)-FA-TEGEC-CL-20/PC** and **HA(270)-FA-HEGEC-CL-20/PC**) were obtained with a drug-to-material ratio of 1:2 (*w*/*w*). Zeta potential measurements of the new complexes (−1.23 mV for **HA(270)-FA-TEGEC-CL-20/PC** and −1.73 mV for **HA(270)-FA-HEGEC-CL-20/PC**) showed alterations compared to the zeta potential values of the materials on their own, suggesting the achievement of drug–material interactions. According to the in vitro dissolution studies carried out in different conditions, novel drug delivery systems (DDSs) were obtained with a variety of characteristics depending on the desired route of administration and, consequently, on the pH of the surrounding environment, thanks to the complexation of phycocyanin with these two new crosslinked materials. Both complexes showed excellent potential for providing a controlled/prolonged release of the active pharmaceutical ingredient (API). They also increased the amount of drug that reach the target location, enabling pH-dependent release. Importantly, as demonstrated by the DPPH free radical scavenging assay, the complexation process, involving freezing and freeze-drying, showed no adverse effects on the antioxidant activity of phycocyanin. This activity was preserved in the two novel materials and followed a concentration-dependent pattern similar to pure PC.

## 1. Introduction

Among glycosaminoglycan derivatives, hyaluronic acid (**HA**, hyaluronan) is one of the most representative [1]. **HA** is generated by the condensation of glucuronic acid and *N*-acetylglucosamine linked by β[1-4] and β[1-3] alternate glycosidic bonds [2]. Thanks to the interaction with the CD44 receptor, a multifunctional transmembrane glycoprotein diffused in almost all human cells, **HA** is implicated in the pericellular coat formation, which was proposed to play a role in the early stages of cell adhesion [3,4]. The ensuing activation of **HA**-CD44 signaling pathways affects several cell functions such as angiogenesis [5], proliferation [6], migration [7], aggregation [8], and adhesion to extracellular matrix (ECM) components [9]. Owing to its capability of binding water, **HA** also regulates a wide range of important physiological functions in the human body such as tissue hydration [10], tissue repair [11], and damping of excessive loads on the joints [12]. Furthermore, **HA** is frequently used as a lubricant supplement in the treatment of diseases involving the osteoarticular and eye districts [13,14]. With the aim to design and synthesize innovative hyaluronan derivatives with enhanced properties in the formulation of drug delivery systems (DDSs), **HA** is still intensively investigated to disclose its biosynthetic pathways and improve its biotechnological production [13,15,16]. Particularly, crosslinked and non-crosslinked **HA** derivatives have been largely employed in the obtainment of biocompatible hydrogels for medical applications [17,18,19,20,21].

Ferulic acid (**FA**) represents a powerful antioxidant cinnamic acid derivative (i.e., 4-hydroxy-3-methoxycinnamic acid) showing free radical scavenging activity [22]. Furthermore, **FA** possesses several physiological functions ranging from antidiabetic activity [23] to anti-inflammatory effects [24,25]. **FA** is also implicated in crosslinking polysaccharides and proteins during cell wall synthesis [26]. Particularly, **FA** residues are attached by an ester bond to the primary alcoholic groups of arabinose side chains in the cell wall arabinoxylan polysaccharides [27,28,29,30,31,32].

**Figure 1:**
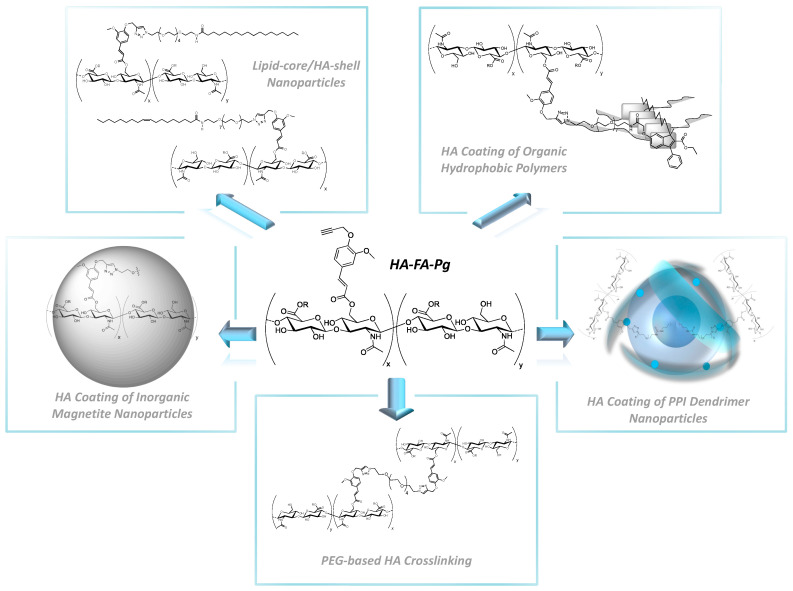
Development and applications of the **HA**-based technology platform employing **HA**-**FA**-**Pg** graft copolymers.

In our laboratories, **FA** has been used as a functionalizing molecule for biocompatible macromolecules [33,34,35]. In particular, **FA** residues bearing propargyl groups were used in the grafting reaction of **HA** macromolecules to afford the resulting hyaluronan derivative **HA**-**FA-Pg** graft copolymers (Figure 1), which were employed in the building of a tri-component polybenzofulvene cylindrical brush through a convergent approach based on a copper(I)-catalyzed azide-alkyne 1,3-dipolar cycloaddition (CuAAC) [36,37].

The coating technique of the polybenzofulvene cylindrical brush with **HA** employing **HA**-**FA**-**Pg** graft copolymers was progressively translated into a well-established technology platform. This allowed the coating of surfaces of different nanostructures to be easily performed in liposomes [38], self-assembling micelles [39], magnetic nanoparticles [40], and poly(propylene imine) (PPI) dendrimers [41]. Moreover, we recently developed a click-chemistry-based crosslinking procedure (click-crosslinking) based on the CuAAC reaction of the clickable propargyl groups of **HA**-**FA**-**Pg** graft copolymers (showing different molar mass and grafting degree values) with a hexa(ethylene glycol) derivative bearing azide groups at both ends, leading to crosslinked **HA** derivatives (i.e., **HA**-**FA**-**HEG**-**CL**) capable of producing interesting hydrogels [42]. The CuAAC crosslinking technique has gained several applications in different fields, especially for the preparation of hydrogels for drug delivery [43]. In this way, this strategy has been revealed as suitable for the synthesis of click-crosslinked viral nanoparticles bearing “clickable” fluorescent crosslinkers at the interface. The resulting drug-conjugated nanoparticles are expected to have potential applications for drug delivery in chemotherapy [44]. Li and coworkers have realized an efficient synthesis of novel crosslinking amino esters that are useful as protein crosslinks for various biological applications [45]. Schellinger and collaborators have employed the CuAAC procedure for the crosslinking of multivalent single chain variable fragments to achieve multivalent and bi-specific immunoconjugates with biological activity, which are potentially useful in pre-targeted radioimmunotherapy and imaging [46]. Furthermore, the click-chemistry-based crosslinking procedure has been developed for the cellulose reticulation to obtain crosslinked material as a model for future works on paper pulp [47].

The results of the previous work performed on the click-crosslinking of **HA**-**FA**-**Pg** graft copolymers with hexa(ethylene glycol) derivative bearing azide groups at both ends stimulated the exploration of more complex cross-linking agents showing a greater length and/or bearing functional moieties, such as aromatic groups of cinnamic acid derivatives.

**Figure 2:**
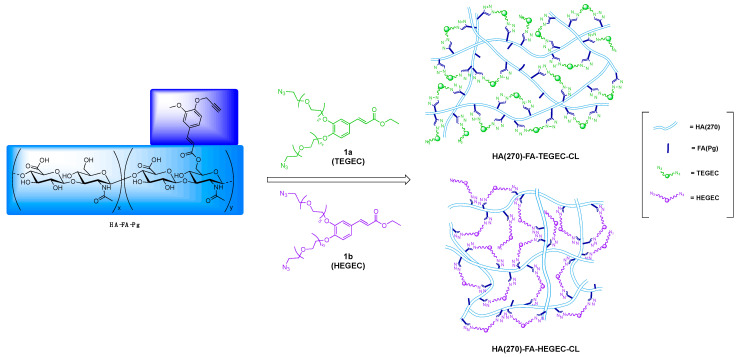
Click-chemistry crosslinking of **HA**-**FA**-**Pg** graft copolymers to obtain crosslinked **HA** derivatives **HA(270)-FA-TEGEC-CL** and **HA(270)-FA-HEGEC-CL**.

Thus, in the present work, clickable crosslinking agents **1a** (**TEGEC**) and **1b** (**HEGEC**) were designed, synthesized, and employed in the CuAAC click-crosslinking coupling of the graft copolymers **HA(270)-FA**-**Pg** showing a medium molar mass value (i.e., Mw = 270 kDa) and different grafting degree values (i.e., 10, 20, and 40%) to obtain crosslinked **HA** derivatives (i.e., **HA(270)-FA-TEGEC-CL** and **HA(270)-FA-HEGEC-CL**) (Figure 2) showing different crosslinking densities.

**Figure 3:**
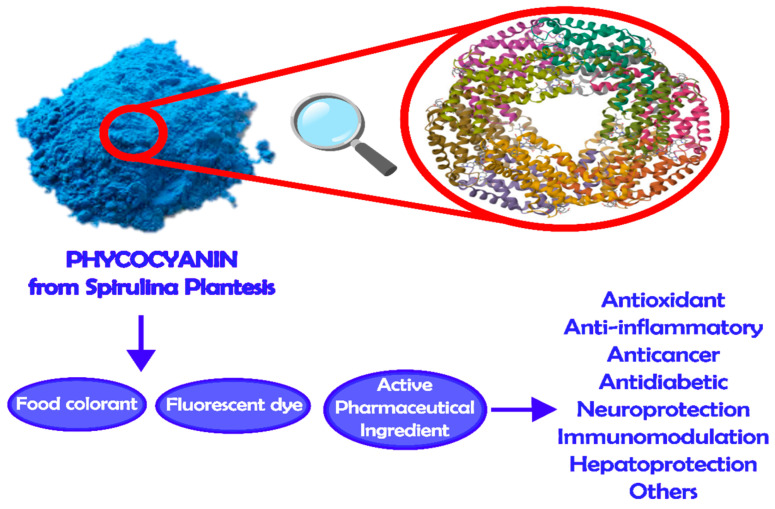
Crystal structure of PC from cyanobacterium SP in the form of the hexamer—image from PDB https://www.rcsb.org/structure/1i7y (accessed on 15 November 2023)—and a schematic overview of the main applications and pharmacological properties of PC.

The obtained crosslinked materials were studied from the point of view of their structure and their aggregation liability to form hydrogels in the water environment. The most promising materials showed interesting loading capability regarding the active pharmaceutical ingredient (API) called phycocyanin (PC), a water-soluble, blue-colored pigment protein present in several blue-green algae species [48]. PC represents the primary bioactive component in the microalga Spirulina Plantesis (SP) [49], which has been suggested by the World Health Organization (WHO) as a possible source of the therapeutic natural chemical [50,51]. PC forms a heterodimer consisting of α (CpcA, C-phycocyanin alpha-subunit gene product) and β (CpcB, C-phycocyanin beta-subunit gene product) subunits [52]. PC’s chromophore is phycocyanobilin (PCB) [53], covalently linked to the apoprotein through a thioether bond. PC incorporates three PCBs linked to Cys-84 of the alpha chain, Cys-82, and 155 of the beta chain, which are shielded from degradation and maintain stability within the protein’s hydrophobic core (Figure 3) [54,55,56].

The PC’s distinctive structure also makes it a flexible tool for biotechnology. Its applications extend to biosensors [57], biocatalysts, and drug delivery systems [58]. In the cosmetic and food industries, PC is favored for its non-toxicity and absence of carcinogenic potential, making it a safe choice for various products, including food coloring [59]. Its pronounced fluorescence also permits the development of fluorescent reagents, probes, and tracers, which are extensively used in immunology, biological engineering, and various scientific disciplines [60]. In addition, PC has several types of pharmacological applications, including anti-inflammatory [61], anticancer [62], neuroprotective [63], hepatoprotective [64], immunomodulatory [65], renoprotective [66], and anti-diabetic properties [67,68,69]. These properties make PC a valuable candidate for several therapeutic applications. One of the foremost attributes of PC is its antioxidant activity [70]. Antioxidants play a pivotal role in safeguarding cells and tissues against oxidative stress, a process intricately linked to numerous chronic diseases and the aging process. PC’s proficiency in neutralizing free radicals and mitigating oxidative stress underscores its potential as a therapeutic agent [71]. Given the potential of PC, our research team has selected this API for several prospective applications [72,73].

Although PC shows the above-mentioned activities, its stability is highly dependent on temperature and pH [74]. PC’s stability is mostly determined by protein structure, and every factor that modifies the stability and structure of proteins has the potential to either delay or speed up PC degradation [75]. Stabilizing agents have been tested for their ability to preserve the native structure of proteins and the color of PC molecules. Polymers, protein crosslinking agents, and preservatives have also been investigated for their potential to protect PC [76,77].

In this context, our investigation focuses on the development and characterization of novel drug delivery systems (DDSs) by exploiting the features of PC in combination with novel crosslinked materials, such as **HA(270)-FA-TEGEC-CL-20** and **HA(270)-FA-HEGEC-CL-20.** By manipulating the interaction between PC and these advanced materials, we aim to create DDSs with controlled and sustained release profiles of PC. This strategic approach aims to improve PC’s performance and optimize its therapeutic potential across several illnesses by customizing medication delivery methods to PC’s unique features, leading to more efficient and focused therapies to improve healthcare.

## 2. Results and Discussion

### 2.1. Synthesis of HA(270)-FA-TEGEC-CL and HA(270)-FA-HEGEC-CL Derivatives

**Scheme 1:**
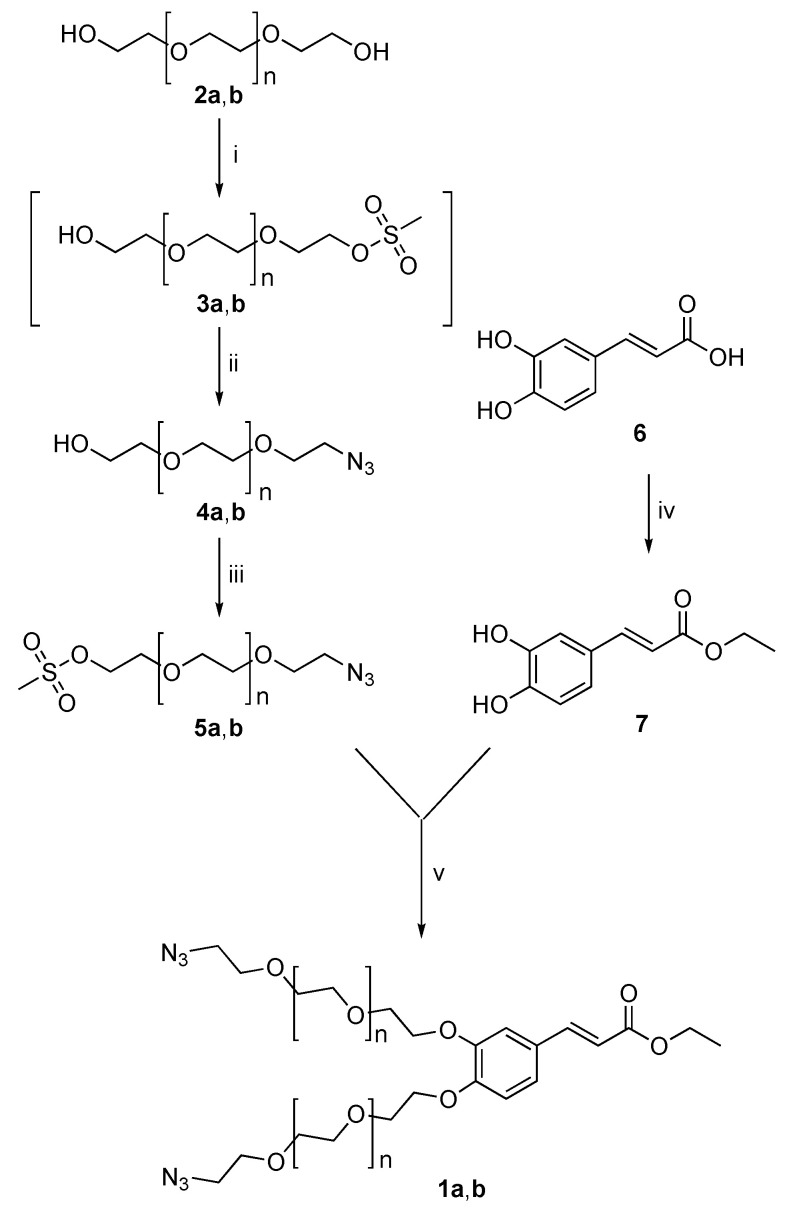
Synthesis of appropriate clickable crosslinking agents **1a** (**TEGEC**) and **1b** (**HEGEC**). **Reagents**: (i) CH_3_SO_2_Cl, TEA, CH_2_Cl_2_; (ii) NaN_3_, DMF, CH_3_CN; (iii) CH_3_SO_2_Cl, TEA, CH_2_Cl_2_; (iv) POCl_3_, EtOH; (v) CsCO_3_, NaI, CH_3_CN. **a**: n = 1; **b**: n = 4.

The synthetic work started with the convergent procedure (Scheme 1) leading to the appropriate clickable crosslinking agents **1a** (**TEGEC**) and **1b** (**HEGEC**).

In particular, the suitable commercially available oligo(ethylene glycol) derivatives [i.e., tri(ethylene glycol) **2a** for compound **1a** and hexa(ethylene glycol) **2b** for compound **1b**] were made to react with mesyl chloride in DCM in the presence of TEA as the base to obtain the corresponding mono-mesylates, **3a**,**b**, which were promptly converted into the corresponding mono-azido derivatives, **4a**,**b**. The latter were activated through a reaction with mesyl chloride in DCM in the presence of TEA as the base to afford the mesylate derivatives **5a**,**b**, which were employed in the alkylation of the catechol groups of ethyl caffeate **7** in the presence of cesium carbonate as the base and sodium iodide as the catalyst to obtain compounds **1a**,**b** (**TEGEC** and **HEGEC**, respectively) in acceptable yields.

**Scheme 2:**
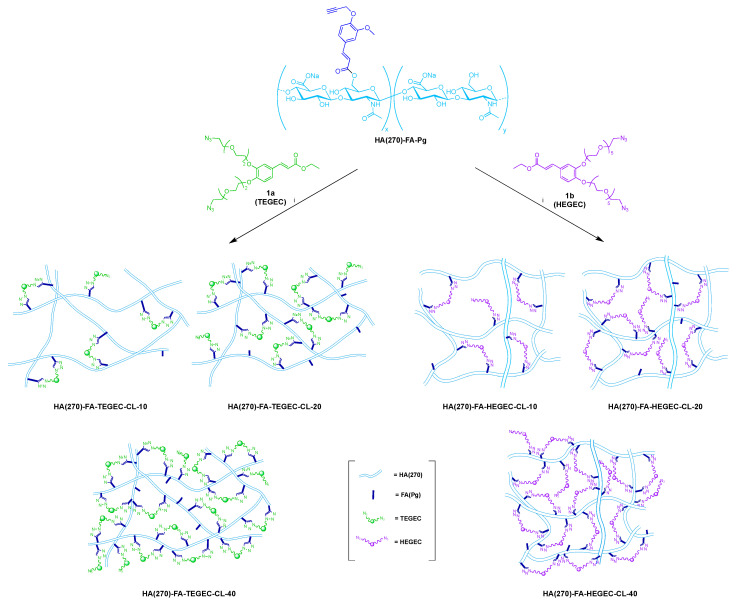
Click-chemistry crosslinking of **HA(270)**-**FA**-**Pg** derivatives showing different grafting degree values with divalent azide derivatives **1a**,**b** leading to **HA(270)-FA-TEGEC-CL** and **HA(270)-FA-HEGEC-CL** materials. **Reagents**: (i) CuSO_4_, sodium ascorbate, *tert*-butanol, H_2_O.

Clickable crosslinking agents **1a** (**TEGEC**) and **1b** (**HEGEC**) were then used in the CuAAC reaction [78,79] with **HA(270)**-**FA**-**Pg** graft copolymers showing different grafting degree values (i.e., 10, 20, and 40%) to obtain crosslinked **HA** derivatives (i.e., **HA(270)-FA-TEGEC-CL** and **HA(270)-FA-HEGEC-CL**) showing different crosslinking densities (Scheme 2).

The click-crosslinking reaction was performed by using three **HA(270)**-**FA**-**Pg** samples showing grafting degree values of 10, 20, and 40% to obtain **HA(270)-FA-TEGEC-CL** and **HA(270)-FA-HEGEC-CL** materials showing different crosslinking densities and crosslinking groups that could affect the flexibility of the **HA(270)** backbone in the crosslinked materials. Therefore, the amounts of divalent azide derivatives **1a**,**b** were planned to induce the exhaustive reaction of the propargyl groups on the ferulate residues grafting the **HA** backbones of **HA(270)-FA**-**Pg** graft copolymers. Furthermore, the catalytic species copper(I) was generated in situ from CuSO_4_ by using sodium ascorbate as the reducing agent to perform the CuAAC dimerization reaction under very mild conditions.

### 2.2. Structure of HA(270)-FA-TEGEC-CL and HA(270)-FA-HEGEC-CL Materials

**Figure 4:**
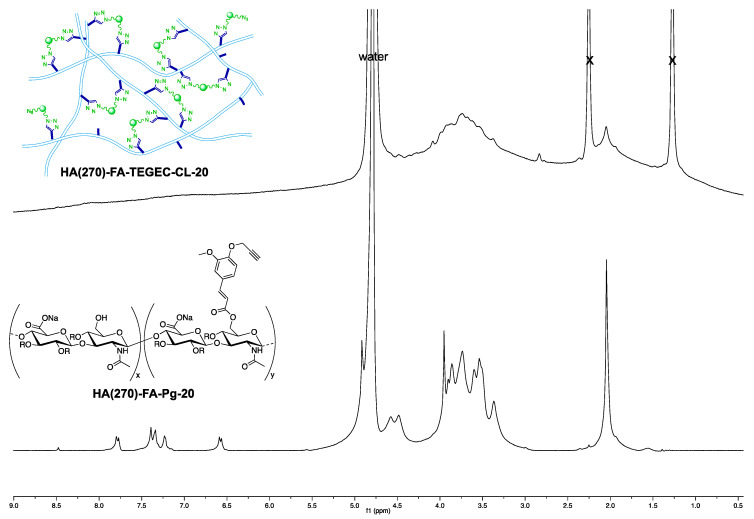
Comparison between the ^1^H NMR (D_2_O, 600 MHz) spectrum, obtained using the crosslinked material **HA(270)-FA-TEGEC-CL-20** (top trace), and that obtained using its corresponding synthetic precursor, the graft copolymer **HA(270)-FA-Pg**-**20** (bottom trace).

The newly synthesized crosslinked materials **HA(270)-FA-TEGEC-CL** and **HA(270)-FA-HEGEC-CL** were subjected to ^1^H NMR spectroscopy characterization using D_2_O as the solvent to obtain information about their structures. In particular, to confirm the occurrence of the click-chemistry crosslinking reaction, the ^1^H NMR spectra of the materials were compared to that of their corresponding synthetic precursors. For instance, the ^1^H NMR spectrum of **HA(270)-FA-TEGEC-CL-20** was compared with that of the corresponding graft copolymer **HA(270)-FA-Pg-20** (Figure 4).

The comparison reported in Figure 4 shows a remarkable line broadening in all the peaks in the spectrum of **HA(270)-FA-TEGEC-CL-20** material (compared to the one visible in the corresponding spectrum of starting **HA(270)-FA-Pg**-**20** graft copolymer) that could easily be explained by the reduced mobility in the crosslinked macromolecules of the material. Unfortunately, because of this line broadening, the peaks in the aromatic region of the spectrum of the crosslinked material were very broad and difficult to interpret in absolute terms. However, the comparison shown in Figure 4 allowed the singlet at around 8 ppm—attributed to the triazole proton—to be detected, confirming the occurrence of the CuAAC reaction.

**Figure 5:**
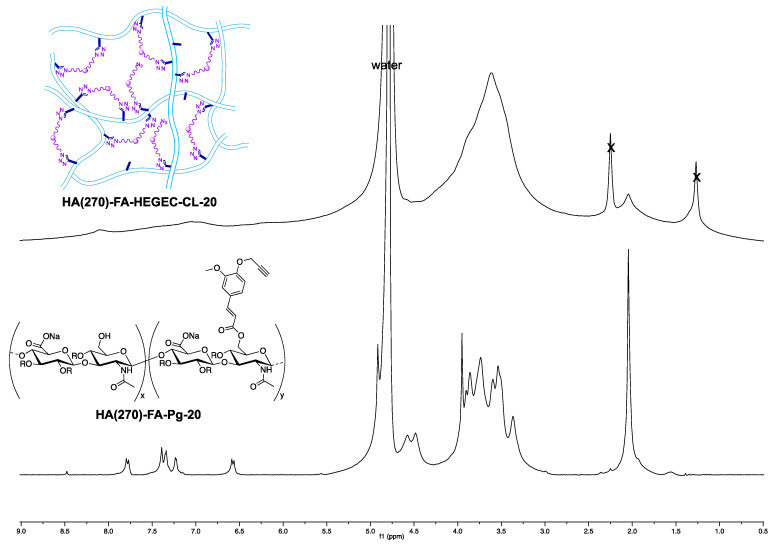
Comparison between the ^1^H NMR (D_2_O, 600 MHz) spectrum, obtained using the crosslinked material **HA(270)-FA-HEGEC-CL-20** (top trace), and that obtained using its corresponding synthetic precursor, the graft copolymer **HA(270)-FA-Pg**-**20** (bottom trace).

Similar results were obtained with all the other crosslinked materials. Figure 5 shows another example, in which the spectrum of crosslinked material **HA(270)-FA-HEGEC-CL-20** (obtained by using **1b** as the crosslinking agent) was compared with that of the corresponding graft copolymer **HA(270)-FA-Pg-20**.

Interestingly, the singlet at around 8 ppm—attributed to the triazole proton—could easily be detected in the spectrum of the crosslinked material **HA(270)-FA-HEGEC-CL-20,** confirming the occurrence of the CuAAC reaction.

### 2.3. Application of HA(270)-FA-TEGEC-CL and HA(270)-FA-HEGEC-CL Crosslinked Materials in the Delivery of Phycocyanin

All the obtained crosslinked materials were preliminarily characterized through dynamic light scattering (DLS) and zeta potential analysis to obtain information on their size, aggregation features, and surface conditions in a water environment. Based on the results of this preliminary characterization study (Table S1, Supplementary Materials), **HA(270)-FA-TEGEC-CL-20** and **HA(270)-FA-HEGEC-CL-20** were selected for the preparation of phycocyanin-loaded materials due to their intermediate size and surface charge characteristics. PC, a water-soluble, blue-colored protein, is a nutraceutical compound with biological activity isolated and/or purified from seaweeds [80]. This protein has garnered a lot of attention in the scientific community due to its numerous health-promoting properties. One of the primary properties of PC is its ability to act as a potent antioxidant [81]. PC’s ability to neutralize free radicals and reduce oxidative stress may contribute to many of its beneficial properties. Additionally, its anti-inflammatory property is another significant feature [67]. Chronic inflammation is associated with numerous diseases, including cardiovascular diseases, autoimmune diseases, and even cancer. We selected this active ingredient, with all of its prospective applications, to utilize the novel crosslinked materials **HA(270)-FA-TEGEC-CL-20** and **HA(270)-FA-HEGEC-CL-20**, and for the preparation and characterization of new DDSs that modify and/or control the release of this protein. This was accomplished by first analyzing the dimensions and surface characteristics of the empty **HA(270)-FA-TEGEC-CL-20** and **HA(270)-FA-HEGEC-CL-20** materials, which displayed average hydrodynamic diameters ranging from about 332–349 nm and roughly equal negative zeta potentials of about −40 mV (Table 1).

**Figure 6:**
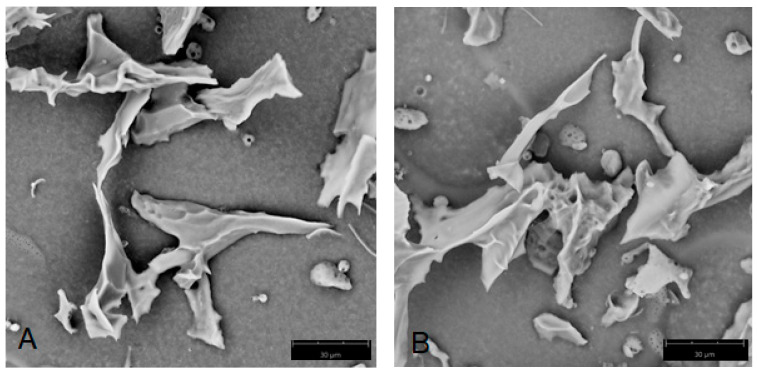
SEM images of the complexes. (**A**) **HA(270)-FA-TEGEC-CL-20/PC**; (**B**) **HA(270)-FA-HEGEC-CL-20/PC**. Size bar is 30 μm.

The two novel material complexes with a drug/material ratio of 1:2 (*w*/*w*), **HA(270)-FA-TEGEC-CL-20/PC** and **HA(270)-FA-HEGEC-CL-20/PC**, have also been characterized. Firstly, the yields of the two production processes were both above 83.5% ± 0.7. Z-average analyses of the two material complexes revealed that they were above the nanometric range, revealing an interaction between PC and the crosslinked materials. We can clearly say that the drug–material interaction has been achieved, since the results of the zeta potential measurements of the new complexes (−1.23 mV for **HA(270)-FA-TEGEC-CL-20/PC** and −1.73 mV for **HA(270)-FA-HEGEC-CL-20/PC**) appeared to be different from the Z-potential values of the crosslinked materials alone. The complementary nature of the combined materials, the crosslinked materials with negative surface charges, and PC with a positive zeta potential allowed the formation of physical bonds, including electrostatic interactions. The experimental DL% of **HA(270)-FA-TEGEC-CL-20/PC** was calculated using UV spectrophotometric analysis, and it was discovered to be equal to 41% p/p (Table 1). The drug incorporation efficiency (EI%) appears to be around 90% p/p. Similarly, **HA(270)-FA-HEGEC-CL-20/PC** exhibits an experimental DL% of 42%. The sizes and shapes of the two complexes were also investigated using scanning electron microscopy (SEM) analysis (Figure 6).

The images show how the two complexes each have an absolutely irregular and quite compact shape. Two different in vitro dissolution studies were carried out to highlight the differences in the release profiles of loaded PC in the new materials compared to the pure API. The evaluation of the effects on the dissolution profiles of the complexes made it possible to appreciate the potential of these materials to create new DDSs.

**Figure 7:**
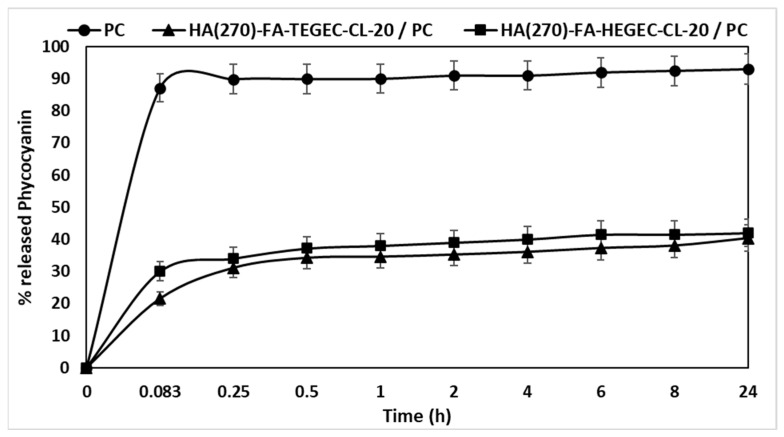
Cumulative percentage of phycocyanin released from **HA(270)-FA-TEGEC-CL-20/PC** and **HA(270)-FA-HEGEC-CL-20/PC** complexes compared with the dissolution profile of free phycocyanin in PBS pH 7.4.

In this regard, a quantity of the two **HA(270)-FA-TEGEC-CL-20/PC** and **HA(270)-FA-HEGEC-CL-20/PC** complexes equivalent to 5.0 mg of pure phycocyanin was placed inside two filter paper bags, and each of them was immersed in a beaker containing 100 mL of PBS for 24 h, maintained under a magnetic stirrer, and placed in a temperature-controlled water bath maintained at 37 °C. From the results obtained in this dissolution study (Figure 7), it was possible to observe that the two **HA(270)-FA-TEGEC-CL-20/PC** and **HA(270)-FA-HEGEC-CL-20/PC** complexes showed dissolution profiles which were not triggered (or instantaneous), like that of the pure PC, but modified in particular by the prolonged/delayed type.

While the percentage of the dissolved phycocyanin reached 90% within the first 30 min due to its high solubility in this medium, the two complexes, **HA(270)-FA-TEGEC-CL-20/PC** and **HA(270)-FA-HEGEC-CL-20/PC**, only released a fraction of the loaded phycocyanin during this initial period. Specifically, **HA(270)-FA-HEGEC-CL-20/PC** released only 37% of the loaded phycocyanin, and **HA(270)-FA-TEGEC-CL-20/PC** released even less. After the full 24 h duration of the experiment, while the pure phycocyanin was almost completely released, the two complexes did not release the entirety of the loaded phycocyanin (i.e., they achieved around 40% PC release). The limited release of phycocyanin from the complexes after 24 h affirms their ability to provide prolonged therapeutic efficacy. This controlled and gradual release is indicative of the complexes’ potential to optimize drug delivery, ensuring sustained bioavailability and therapeutic impact over an extended timeframe. The distinctive release profiles observed in these complexes not only highlight their technological significance, but also underscore their potential application in achieving sustained and controlled drug release for enhanced therapeutic outcomes.

**Figure 8:**
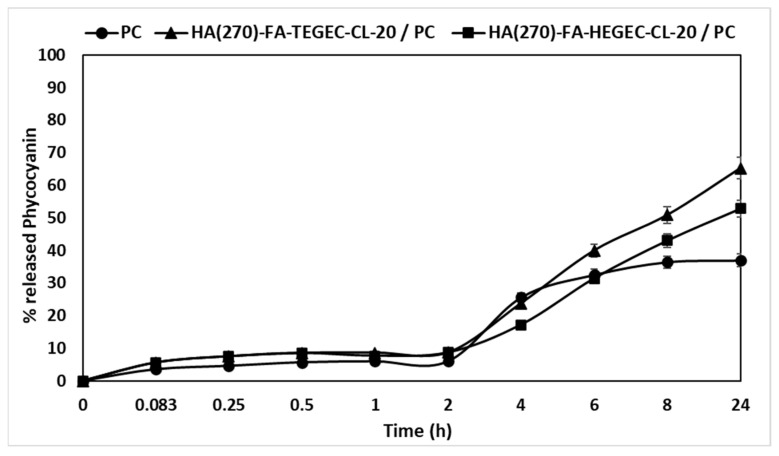
Cumulative percentage of phycocyanin released from **HA(270)-FA-TEGEC-CL-20/PC** and **HA(270)-FA-HEGEC-CL-20/PC** complexes compared with the dissolution profile of free phycocyanin in a pH-changing environment.

Then, we evaluated a different setup for the in vitro release study. Filter paper bags were filled with **HA(270)-FA-TEGEC-CL-20/PC** and **HA(270)-FA-HEGEC-CL-20/PC** complexes, each containing 5.0 mg of pure phycocyanin. They were immersed in a beaker containing 100 mL of 0.1 N HCl, maintained under a magnetic stirrer, and placed in a temperature-controlled water bath maintained at 37 °C. After 2 h, the pH value was raised to 6.8 and remained constant for 22 h. The new experimental setting aimed to replicate the conditions of a pharmaceutical form taken orally [42]. In this experimental set-up (as shown in Figure 8), phycocyanin’s release profile was sensitive to the environment pH. In fact, during the first 2 h at the acidic pH, the amount of PC released was less than 10%.

It was only after the pH jump that the released amount gradually increased, reaching a value of 37% at the end of the experiment. On the other hand, the amount of phycocyanin released from the two complexes at 24 h (65% for **HA(270)-FA-TEGEC-CL-20/PC** and 53% for **HA(270)-FA-HEGEC-CL-20/PC**) was significantly higher than the amount released from the pure drug. These results suggest that the complexation of phycocyanin with these two new materials enables the development of novel DDSs exhibiting various properties and behaviors. In fact, the incorporation with these new materials allows for a tunable response, enabling the DDSs to adapt to different pH environments. This pH responsiveness is particularly crucial in the context of drug delivery, where variations in pH level are characteristic of distinct anatomical sites or physiological compartments. By leveraging the pH sensitivity of the complexes, it becomes possible to modulate drug release rates, ensuring optimal therapeutic efficacy based on the specific requirements of the targeted route of administration. Both approaches demonstrate excellent potential for ensuring a controlled/prolonged release of the active ingredient, avoiding the burst effect associated with pure phycocyanin, and ensuring a pH-dependent release by boosting the amount of drug that can reach the target site.

The antioxidant features of the material complexes **HA(270)-FA-TEGEC-CL-20/PC** and **HA(270)-FA-HEGEC-CL-20/PC** were tested at different concentrations by evaluating their free radical scavenging properties through a DPPH assay.

**Figure 9:**
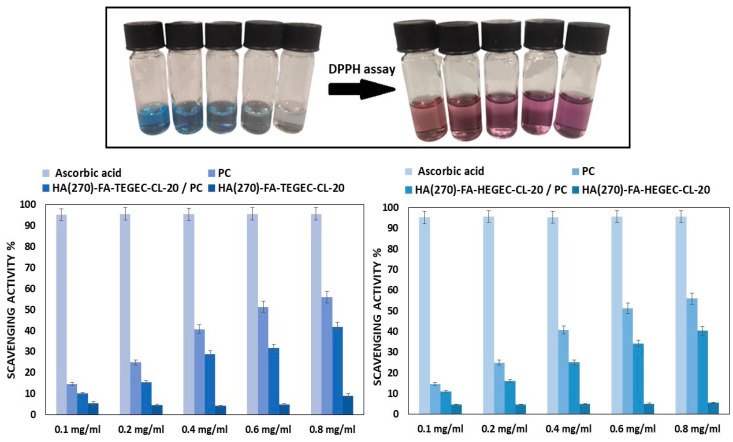
Radical scavenging activities of **HA(270)-FA-TEGEC-CL-20/PC** and **HA(270)-FA-HEGEC-CL-20/PC** were measured through DPPH assay.

The results shown in Figure 9 demonstrate that, after 20 min of incubation, the samples showed dose-dependent scavenging activities. The PC’s ability to eliminate DPPH free radicals increased as the concentration increased. As evident from the data, the complexation process (freezing and freeze-drying) did not affect the phycocyanin’s antioxidant activity, which was preserved in the two new materials produced with the same concentration-dependent pattern as pure PC. The same test was also conducted on the crosslinked materials, which did not show any significant radical scavenging activity. Additionally, a positive reference control (ascorbic acid) was tested at the same dosage to validate the methodology employed.

## 3. Conclusions

Based on the encouraging results obtained, the click-chemistry crosslinking (click-crosslinking) procedure, previously developed to obtain crosslinked **HA** derivatives useful in the development of hydrogels, was further explored. In particular, the clickable propargyl groups of **HA-FA-Pg** graft copolymers showing medium molecular weight values were exploited during the CuAAC coupling (in very mild conditions with the presence of extremely low amounts of copper(I) catalyst) with new clickable crosslinking agents **1a** (**TEGEC**) and **1b** (**HEGEC**) to obtain crosslinked **HA** derivatives showing different crosslinking densities. This work was conceived as another example of the possible applications of our technology platform based on **HA-FA-Pg** graft copolymers. Two short series of crosslinked materials (i.e., **HA(270)-FA-TEGEC-CL** and **HA(270)-FA-HEGEC-CL**) showing different crosslinking densities were synthesized and investigated from the point of view of their structure and aggregation liability to form hydrogels in a water environment.

Using the unique characteristics of PC together with novel crosslinked materials like **HA(270)-FA-TEGEC-CL-20** and **HA(270)-FA-HEGEC-CL-20**, we developed and characterized new drug delivery systems (DDSs). We are therefore able to say that the complexation of phycocyanin with these two novel materials enables the development of novel DDSs that display a variety of properties and behaviors depending on the desired route of administration and, consequently, depending on the pH of the surrounding environment. These DDSs are designed to modify PC release, enhancing its efficacy in treating a range of health issues.

## 4. Materials and Methods

### 4.1. Synthesis and Characterization

All reagents and solvents were obtained from Sigma-Aldrich (Saint Louis, MO, USA) and were used without further purification. Merck TLC (Rahway, NJ, USA) aluminum sheets and silica gel 60 F_254_ were employed for TLC. NMR spectra were recorded with either a Varian Mercury 300, a Bruker DRX-400 AVANCE (Billerica, MA, USA), a Bruker AVANCE III 500, or a Bruker AMX-600 AVANCE spectrometer in the indicated solvents. The chemical shift (δ) values are reported in ppm, and those of the H-H coupling constants (*J*) in Hz. Any mass spectrometry measurements were performed with an Agilent (Santa Clara, CA, USA) 1100 LC/MSD employing an electrospray source.

**2-(2-(2-Hydroxyethoxy)ethoxy)ethyl methanesulfonate (3a).**

To a cooled (0 °C) solution of commercially available tri(ethylene glycol) **2a** (1.0 g, 6.66 mmol) in dry CH_2_Cl_2_ (20 mL), TEA (0.80 mL, 5.99 mmol) and mesyl chloride (0.50 mL, 5.99 mmol) were added in sequence, dropwise. The reaction mixture, after being stirred at room temperature for 1 h under a nitrogen atmosphere, was treated with a saturated solution of NH_4_Cl. The organic layer was then dried over anhydrous sodium sulfate, filtered, and concentrated under reduced pressure to obtain an oily residue containing compound **3a** as a reactive intermediate without further purifications.

**17-Hydroxy-3,6,9,12,15-pentaoxaheptadecyl methanesulfonate (3b).**

To a cooled (0 °C) solution of commercially available hexa(ethylene glycol) **2b** (1.0 g, 3.54 mmol) in dry CH_2_Cl_2_ (20 mL), TEA (0.50 mL, 3.19 mmol) and mesyl chloride (0.25 mL, 3.19 mmol) were added in sequence, dropwise. After stirring at room temperature for 1 h under a nitrogen atmosphere, the reaction mixture was treated with a saturated solution of NH_4_Cl, and the organic layer was dried over anhydrous sodium sulfate, filtered, and evaporated under reduced pressure to obtain an oily residue containing compound **3b** as a reactive intermediate without further purifications.

**2-(2-(2-Azidoethoxy)ethoxy)ethan-1-ol (4a).**

To a solution of the organic residue containing compound **3a** (approximately 6.66 mmol) in dry CH_3_CN (15 mL) and dry DMF (5.0 mL), sodium azide (2.6 g, 40 mmol) was added, and the mixture was heated to reflux overnight under a nitrogen atmosphere. The reaction mixture was then treated with a saturated solution of NH_4_Cl, and the organic layer was extracted with dichloromethane. The combined extracts were dried over anhydrous sodium sulfate, filtered, and the solvent was removed under reduced pressure to give an oily residue which, after purification through flash chromatography using ethyl acetate as eluent, yielded the compound **4a** (463 mg, yield 40%) as a pale yellow oil. ^1^H NMR (400 MHz, CDCl_3_): 3.38 (m, 2H), 3.57–3.75 (m, 10H). MS (ESI): *m*/*z* 198.0 [M + Na^+^].

**17-Azido-3,6,9,12,15-pentaoxaheptadecan-1-ol (4b).**

To a solution of organic residue containing compound **3b** (approximately 3.54 mmol) in dry CH_3_CN (12 mL) and dry DMF (4.0 mL), sodium azide (1.4 g, 21.2 mmol) was added, and the mixture was heated to reflux overnight under a nitrogen atmosphere. The reaction mixture was then treated with a saturated solution of NH_4_Cl, and the organic layer was extracted with dichloromethane. The combined extracts were dried over anhydrous sodium sulfate, filtered, and concentrated under reduced pressure to give an oily residue, which was purified through flash chromatography using ethyl acetate-methanol (9:1) as the eluent to give the expected compound **4b** (396 mg, yield 36%) as a pale yellow oil. MS (ESI): *m*/*z* 330.1 [M + Na^+^].

**2-(2-(2-Azidoethoxy)ethoxy)ethyl methanesulfonate (5a).**

To a cooled (0 °C) solution of compound **4a** (463 mg, 2.64 mmol) in dry CH_2_Cl_2_ (20 mL), TEA (1.5 mL, 10.6 mmol), and mesyl chloride (1.2 mL, 15.9 mmol) were added dropwise, in sequence. The reaction mixture, after being stirred overnight at room temperature under a nitrogen atmosphere, was treated with a saturated solution of NH_4_Cl. The organic layer was then dried over anhydrous sodium sulfate, filtered, and the solvent was evaporated in vacuo. The resulting residue was purified through flash chromatography, with ethyl acetate-petroleum ether (4:6) as the eluent, to give the expected compound **5a** (629 mg, yield 94%) as a yellow oil. ^1^H NMR (400 MHz, CDCl_3_): 3.06 (s, 3H), 3.38 (t, *J* = 4.9, 2H), 3.57–3.75 (m, 6H), 3.77 (t, *J* = 4.6, 2H), 4.37 (t, *J* = 4.5, 2H). MS (ESI): *m*/*z* 276.0 [M + Na^+^].

**17-Azido-3,6,9,12,15-pentaoxaheptadecyl methanesulfonate (5b).**

To a cooled (0 °C) solution of compound **4b** (396 mg, 1.29 mmol) in dry CH_2_Cl_2_ (10 mL), TEA (0.72 mL, 5.16 mmol), and mesyl chloride (0.6 mL, 7.73 mmol) were added dropwise, in sequence. The reaction mixture, after being stirred at room temperature under a nitrogen atmosphere for 1.5 h, was treated with a saturated solution of NH_4_Cl. The organic layer was then dried over anhydrous sodium sulfate, filtered, and concentrated under reduced pressure. The resulting oily residue was purified through flash chromatography, using ethyl acetate as the eluent, to give the expected compound **5b** (454 mg, yield 91%) as a yellow oil. ^1^H NMR (300 MHz, CDCl_3_): 3.22 (s, 3H), 3.52 (d, *J* = 4.6, 2H), 3.75–3.84 (m, 18H), 3.87–3.92 (m, 2H), 4.48–4.53 (m, 2H). MS (ESI): *m*/*z* 408.1 [M + Na^+^].

**Ethyl (*E*)-3-(3,4-dihydroxyphenyl)acrylate (7)** [82].

To a solution of commercially available caffeic acid **6** (1.0 g, 5.55 mmol) in dry EtOH (20 mL), phosphorus(V) oxychloride (0.76 mL, 8.33 mmol) was added dropwise. After stirring to reflux overnight under a nitrogen atmosphere, the reaction mixture was concentrated under reduced pressure, and the organic residue was partitioned between dichloromethane and water. The combined extracts were dried over anhydrous sodium sulfate, filtered, and the solvent was removed in vacuo. Purification of the crude product through flash chromatography with petroleum ether-ethyl acetate (7:3) as the eluent gave compound **7** (660 mg, yield 57%) as an off-white solid. ^1^H NMR (300 MHz, CD_3_OD): 1.30 (t, *J* = 7.1, 3H), 4.21 (q, *J* = 7.1, 2H), 6.24 (d, *J* = 15.9, 1H), 6.77 (d, *J* = 8.1, 1H), 6.93 (dd, *J* = 8.2, 2.0, 1H), 7.03 (d, *J* = 2.0, 1H), 7.53 (d, *J* = 15.9, 1H). MS (ESI): *m*/*z* 231.1 [M + Na^+^].

**Ethyl (*E*)-3-(3,4-bis(2-(2-(2-azidoethoxy)ethoxy)ethoxy)phenyl)acrylate (1a).**

To a solution of compound **7** (0.26 g, 1.25 mmol) in dry CH_3_CN (25 mL), Cs_2_CO_3_ (1.6 g, 4.98 mmol), NaI (0.37 g, 2.49 mmol), and compound **5a** (0.63 g, 2.49 mmol) were added. After stirring to reflux for 3 h under a nitrogen atmosphere, the reaction mixture was concentrated under reduced pressure, and the organic residue was partitioned between dichloromethane and a 1 N HCl solution. The combined extracts were dried over anhydrous sodium sulfate, filtered, and the solvent was removed in vacuo. The resulting oily residue was purified through flash chromatography, using petroleum ether-ethyl acetate (4:6 *v*/*v*) as the eluent, to give the expected compound **1a** (400 mg, yield 62%) as a pale yellow oil. ^1^H NMR (500 MHz, MeOD): 1.31 (t, *J* = 7.1, 3H), 3.33–3.36 (m, 4H), 3.64–3.68 (m, 8H), 3.72–3.75 (m, 4H), 3.85–3.88 (m, 4H), 4.18–4.24 (m, 6H), 6.38 (d, *J* = 15.9, 1H), 6.99 (d, *J* = 8.4, 1H), 7.16 (dd, *J* = 8.4, 2.0, 1H), 7.26 (d, *J* = 2.0, 1H), 7.60 (d, *J* = 15.9, 1H). MS (ESI): *m*/*z* 545.2 [M + Na^+^].

**Ethyl (*E*)-3-(3,4-bis((17-azido-3,6,9,12,15-pentaoxaheptadecyl)oxy)phenyl)acrylate (1b).**

To a solution of compound **7** (86 mg, 0.41 mmol) in dry CH_3_CN (15 mL), Cs_2_CO_3_ (534 mg, 1.64 mmol), NaI (124 mg, 0.83 mmol), and compound **5b** (318 g, 0.83 mmol) were added. After stirring to reflux under a nitrogen atmosphere for 3 h, the reaction mixture was concentrated under reduced pressure, and the organic residue was partitioned between dichloromethane and a 1 N HCl solution. The combined extracts were dried over anhydrous sodium sulfate, filtered, and concentrated under reduced pressure. The resulting oily residue was purified through flash chromatography, with ethyl acetate-methanol (95:5) as the eluent, to give the expected compound **1b** (128 mg, yield 41%) as a yellow oil. ^1^H NMR (300 MHz, MeOD): 1.35 (t, *J* = 7.1, 3H), 3.37–3.42 (m, 4H), 3.62–3.72 (m, 32H), 3.76 (m, 4H), 3.90 (m, 4H), 4.22–4.30 (m, 6H), 6.44 (d, *J* = 15.9, 1H), 7.05 (d, *J* = 8.4, 1H), 7.21 (dd, *J* = 8.4, 2.0, 1H), 7.31 (d, *J* = 2.0, 1H), 7.64 (d, *J* = 15.9, 1H). MS (ESI): *m*/*z* 809.8 [M + Na^+^].

### 4.2. The General Procedure of Click-Chemistry Crosslinking of HA(270)-FA-Pg Graft Copolymers

A 10 mL flask was charged, under an inert atmosphere, with *tert*-butanol (2.0 mL), water (2.0 mL), and a solution of CuSO_4_ pentahydrate (12.5 mg, 0.050 mmol) in 0.50 mL of water, and a 1 M solution of sodium ascorbate in water (0.50 mL) was then added. Subsequently, 1.0 mL of the resulting mixture was used as the catalyst. A mixture of **1a**,**b** (see the amounts below) and the appropriate **HA(270)**-**FA**-**Pg** graft copolymer (250 mg) [42] in *tert*-butanol (25 mL) and water (25 mL) was treated with the catalyst solution (1.0 mL), and the reaction mixture was stirred overnight at room temperature and then concentrated under reduced pressure. Purification of the gel residue by washing with acetone gave the corresponding crosslinked materials, which were dried under reduced pressure.

**HA(270)-FA-TEGEC-CL-10 material**

This crosslinked **HA** derivative was prepared using the above general procedure of **HA(270)-FA-Pg-10** (250 mg) [42] and crosslinking derivative **1a** (15.5 mg, 0.030 mmol) to obtain **HA(270)-FA-TEGEC-CL-10** material (265 mg) as an off-white solid. ^1^H NMR (600 MHz, D_2_O): Figure S1.

**HA(270)-FA-TEGEC-CL-20 material**

This crosslinked **HA** derivative was prepared using the above general procedure of **HA(270)-FA-Pg-20** (250 mg) [42] and crosslinking derivative **1a** (36 mg, 0.069 mmol) to obtain **HA(270)-FA-TEGEC-CL-20** material (268 mg) as a pale yellow solid. ^1^H NMR (600 MHz, D_2_O): Figure 4.

**HA(270)-FA-TEGEC-CL-40 material**

This crosslinked **HA** derivative was prepared using the above general procedure of **HA(270)-FA-Pg-40** (250 mg) [42] and crosslinking derivative **1a** (64 mg, 0.123 mmol) to obtain **HA(270)-FA-TEGEC-CL-40** material (287 mg) as a pale yellow solid.

**HA(270)-FA-HEGEC-CL-10 material**

This crosslinked **HA** derivative was prepared using the above general procedure of **HA(270)-FA-Pg-10** (250 mg) [42] and crosslinking derivative **1b** (29 mg, 0.040 mmol) to obtain **HA(270)-FA-HEGEC-CL-10 material** (256 mg) as a pale yellow solid. ^1^H NMR (600 MHz, D_2_O): Figure S2.

**HA(270)-FA-HEGEC-CL-20 material**

This crosslinked HA derivative was prepared using the above general procedure of **HA(270)-FA-Pg-20** (250 mg) [42] and crosslinking derivative **1b** (54 mg, 0.069 mmol) to obtain **HA(270)-FA-HEGEC-CL-20 material** (246 mg) as a pale yellow solid. ^1^H NMR (600 MHz, D_2_O): Figure 5.

**HA(270)-FA-HEGEC-CL-40 material**

This crosslinked **HA** derivative was prepared using the above general procedure of **HA(270)-FA-Pg-40** (250 mg) [42] and crosslinking derivative **1b** (97 mg, 0.123 mmol) to obtain **HA(270)-FA-HEGEC-CL-40 material** (302 mg) as a pale yellow solid. ^1^H NMR (600 MHz, D_2_O): Figure S3.

### 4.3. Preparation of Phycocyanin-Loaded HA(270)-FA-TEGEC-CL-20 and HA(270)-FA-HEGEC-CL-20 Materials

An exactly weighted quantity (i.e., 30 mg) of **HA(270)-FA-TEGEC-CL-20** was dispersed in 15 mL of water (concentration: 2.0 mg/mL) and left to stir with a magnetic stirrer for 2 h. Simultaneously, a solution of phycocyanin (PC) in water at a concentration of 3.0 mg/mL (15 mg of PC in 5.0 mL of water) was prepared. Subsequently, the PC solution was added dropwise to the material dispersion, and the resulting dispersion was left to stir with a magnetic stirrer for 2 h. Finally, the sample was frozen and then freeze-dried. The same procedure was used to prepare the complex between **HA(270)-FA-HEGEC-CL-20** and PC. After the PC–material complex production, the percentage yield of the process (%Y) ± SD (mean ± SD, n = 3) was calculated as:

$$\%Y=\frac{Mass\;of\;the\;total\;complex\;obtained}{Mass\;of\;the\;active\;ingredient\;and\;polymer\;in\;the\;complex}\times 100$$

### 4.4. Drug Loading Evaluation

The experimental drug loading (experimental DL%) ± SD (mean ± SD, n = 3) was calculated according to the formula reported below [83], and the amount of PC loaded into the material complex was assessed spectrophotometrically using a UV Jasco V760 spectrophotometer, reading at the maximum wavelength (λ max) of the PC in distilled water of 616 nm (cuvette 1.0 mL, optical path 10 mm).

$$experimental\;DL\%=\frac{Mass\;of\;the\;drug\;in\;the\;polymeric\;complex}{Mass\;of\;the\;polymeric\;complex}\times 100$$

The incorporation efficiency (EI%) was determined by accounting for the embodied PC within its respective complex (experimental DL%) and the amount introduced in the complex’s preparation (theoretical DL%).

$$EI\%=\frac{experimental\;DL\%}{theoretical\;DL\%}\times 100$$

### 4.5. Dynamic Light Scattering and Zeta Potential Measurements

Hydrodynamic diameters, polydispersity indexes (PDI), and zeta potentials were measured by using a Malvern Zetasizer NanoZS instrument (Worcestershire, UK) equipped with a 632.8 nm laser fixed at 173°. The samples (all the polymer materials produced and the two PC–material complexes) were dispersed in ultrapure water (1.0 mg/mL) and analyzed after syringe filtration (cut-off 5 μm) at 25 °C. The mean hydrodynamic diameter and the polydispersity index (PDI) were obtained by evaluating the cumulants of the correlation function.

The zeta potential (mV) was calculated from electrophoretic mobility by using the Smoluchowski relationship and assuming K_a_ > 1 (where K and a are the Debye–Hückel parameter and the particle radius, respectively).

### 4.6. Scanning Electron Microscopy

The complexes’ size and morphology were measured through scanning electron microscopy (SEM) using Phenom ProXSEM (Alfatest, Rome, Italy). The SEM analysis was performed with an electron-accelerating voltage of 15 kV. Samples were mounted on aluminum stubs with double-adhesive carbon tape and dried under a vacuum (0.1 Torr). All the analyses were performed at 25.0 ± 0.1 °C.

### 4.7. In Vitro Dissolution Studies

Amounts of the two **HA(270)-FA-TEGEC-CL-20/PC** and **HA(270)-FA-HEGEC-CL-20/PC** complexes corresponding to 5.0 mg of pure phycocyanin were placed inside two filter paper bags (cut-off 0.45 μm), and each of them was immersed in a beaker containing 100 mL of PBS. In each beaker, a magnetic stirrer was placed to ensure constant agitation of the medium, and the beakers were placed inside a temperature-controlled water bath at 37 °C for 24 h. Sampling was conducted by withdrawing 1.0 mL of solution at scheduled time intervals (5 min, 15 min, 30 min, 1 h, 2 h, 4 h, 6 h, 8 h, and 24 h) and replacing it with the same volume of fresh medium. The samples were analyzed spectrophotometrically using a UV Jasco V760 spectrophotometer (cuvette 1.0 mL, optical path 10 mm). The experiments were carried out in triplicate [84].

Amounts of the two **HA(270)-FA-TEGEC-CL-20/PC** and **HA(270)-FA-HEGEC-CL-20/PC** complexes corresponding to 5.0 mg of pure phycocyanin were placed inside two filter paper bags (cut-off 0.45 μm), and each of them was immersed in a beaker containing 100 mL of 0.1 N HCl. In each beaker, a magnetic stirrer was placed to ensure constant agitation of the medium, and the beakers were placed inside a temperature-controlled water bath at 37 °C. After 2 h, the pH of the systems was increased to 6.8 by adding NaOH 1 M. This pH was maintained for the following 22 h. Sampling was conducted by withdrawing 1.0 mL of solution at scheduled time intervals (5 min, 15 min, 30 min, 1 h, 2 h, 4 h, 6 h, 8 h, and 24 h) and replacing it with the same volume of fresh medium. The samples were analyzed spectrophotometrically using a UV Jasco V760 spectrophotometer (cuvette 1.0 mL, optical path 10 mm) [83]. The experiments were carried out in triplicate.

### 4.8. DPPH Free Radical Scavenging Assay

The DPPH assay was performed to evaluate the antioxidant activity of phycocyanin and the two **HA(270)-FA-TEGEC-CL-20/PC** and **HA(270)-FA-HEGEC-CL-20/PC** complexes. The tested concentrations were as follows: 0.1, 0.2, 0.4, 0.6, and 0.8 mg/mL of PC. Phycocyanin was dissolved in MilliQ water (concentration 1.0 mg/mL), and the solution was stirred with a magnetic stirrer for 2 h. Subsequently, it was filtered using a 0.2 mm RC filter, and serial dilutions were made to obtain the final test concentrations. Next, 10 mg of both complexes were dissolved in a volume of water to achieve the required concentrations, and the solutions were stirred with a magnetic stirrer for 2 h. Then, the samples were filtered by using a 0.2 mm RC filter. The same procedure was employed to assess the potential antioxidant activity of the **HA(270)-FA-TEGEC-CL-20** and **HA(270)-FA-HEGEC-CL-20** crosslinked materials. Additionally, the same assay was performed using the same concentrations of ascorbic acid as a positive control. To each sample, 1.0 mL of a 0.1 mM ethanolic DPPH solution was added. The vials were kept in the dark for 20 min and the samples were analyzed spectrophotometrically using a UV Jasco V760 spectrophotometer (cuvette 1.0 mL, optical path 10 mm) at a wavelength of 524 nm [85]. The percentage of radical scavenging activity was calculated using the following equation:

$$Radical\;scavenging\;activity\;\%=\frac{\left[\left(Abs\;of\;Blank-Abs\;of\;Control\right)-Abs\;of\;Sample\right]}{\left(Abs\;of\;Blank-Abs\;of\;Control\right)}$$

## Figures and Tables

**Table 1:** Z-average, PDI, zeta potential, and drug loading % of empty and phycocyanin-loaded materials.

Sample	Z-Average (nm)	PDI	Zeta Potential (mV)	DL% (*p*/*p*)
**HA(270)-FA-TEGEC-CL-20**	332.5 ± 22.6	0.338 ± 0.012	−39.1 ± 0.7	/
**HA(270)-FA-HEGEC-CL-20**	348.6 ± 18.4	0.279 ± 0.015	−41.5 ± 0.5	/
**HA(270)-FA-TEGEC-CL-20/PC**	/	/	−1.23 ± 0.07	41% ± 2
**HA(270)-FA-HEGEC-CL-20/PC**	/	/	−1.73 ± 0.03	42% ± 1

## Data Availability

All data and materials are available on request from the corresponding author. The data are not publicly available due to ongoing researches using a part of the data.

## Supplementary Materials

The following supporting information can be downloaded at: https://www.mdpi.com/article/10.3390/gels10020091/s1. Figure S1: 1H NMR (D2O, 600 MHz) spectrum of the crosslinked material HA(270)-FA-TEGEC-CL-10; Figure S2: 1H NMR (D2O, 600 MHz) spectrum of the crosslinked material HA(270)-FA-HEGEC-CL-10; Figure S3: 1H NMR (D2O, 600 MHz) spectrum of the crosslinked material HA(270)-FA-HEGEC-CL-40; Table S1: Z-average, PDI, and zeta-potential, of all the crosslinked materials produced (HA(270)-FA-TEGEC-CL-10, HA(270)-FA-TEGEC-CL-20, HA(270)-FA-TEGEC-CL-40, HA(270)-FA-HEGEC-CL-10, HA(270)-FA-HEGEC-CL-20, and HA(270)-FA-HEGEC-CL-40).
