# Supplementary material for: Cross-Linked Hyaluronan Derivatives in the Delivery of Phycocyanin
Source: Gels. 2024 Jan 25;10(2):91. doi: 10.3390/gels10020091 (PMC10887560; doi:10.3390/gels10020091)
Supplement: Supplementary file 1 [file gels-10-00091-s001.zip › gels-2793491-supplementary.pdf]

Contents:

^{1}H NMR spectrum of the crosslinked material **HA(270)-FA-TEGEC-CL-10** page 2

^{1}H NMR spectrum of the crosslinked material **HA(270)-FA-HEGEC-CL-10** page 3

^{1}H NMR spectrum of the crosslinked material **HA(270)-FA-HEGEC-CL-40** page 4

Table of Z-average, PDI, and zeta-potential of all the crosslinked materials obtained page 5

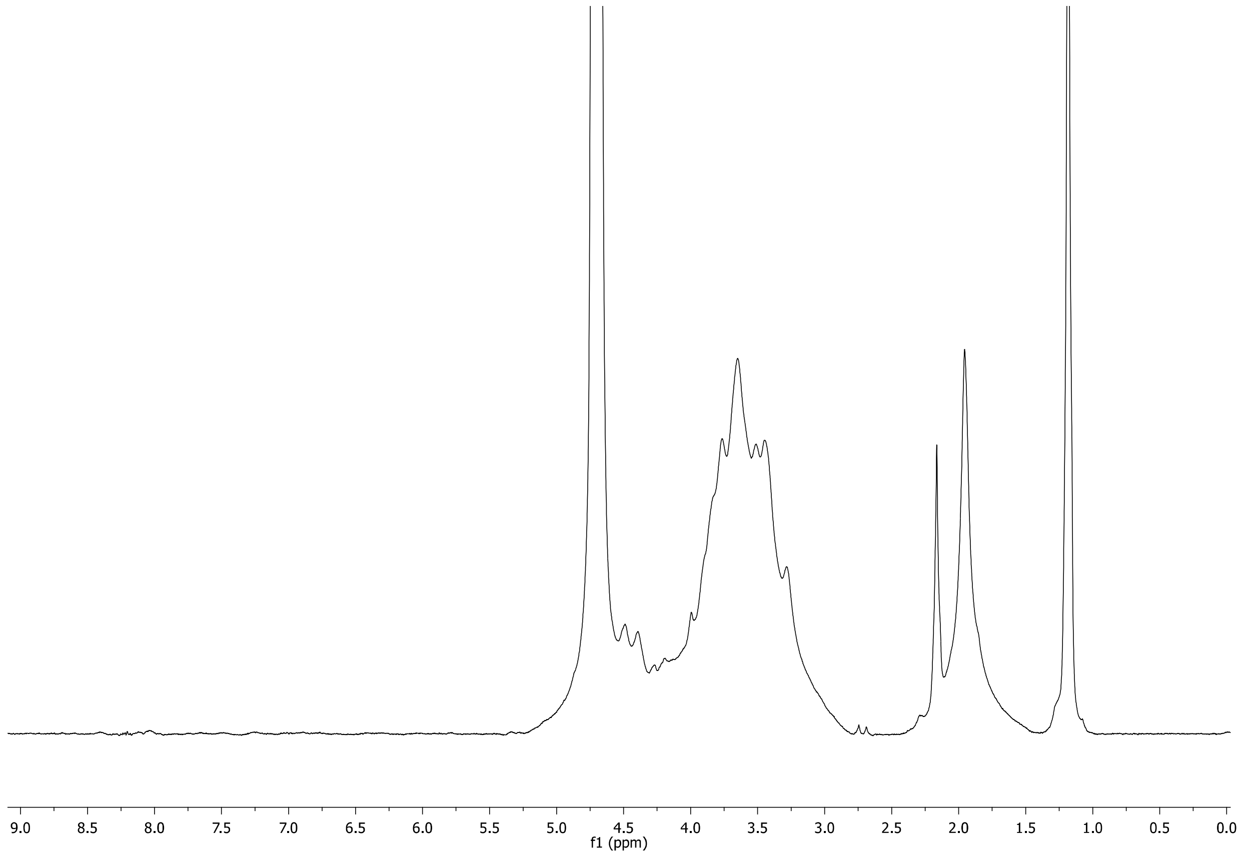

Figure S1. ^{1}H NMR (D_2O, 600 MHz) spectrum of the crosslinked material **HA(270)-FA-TEGEC-CL-10**.

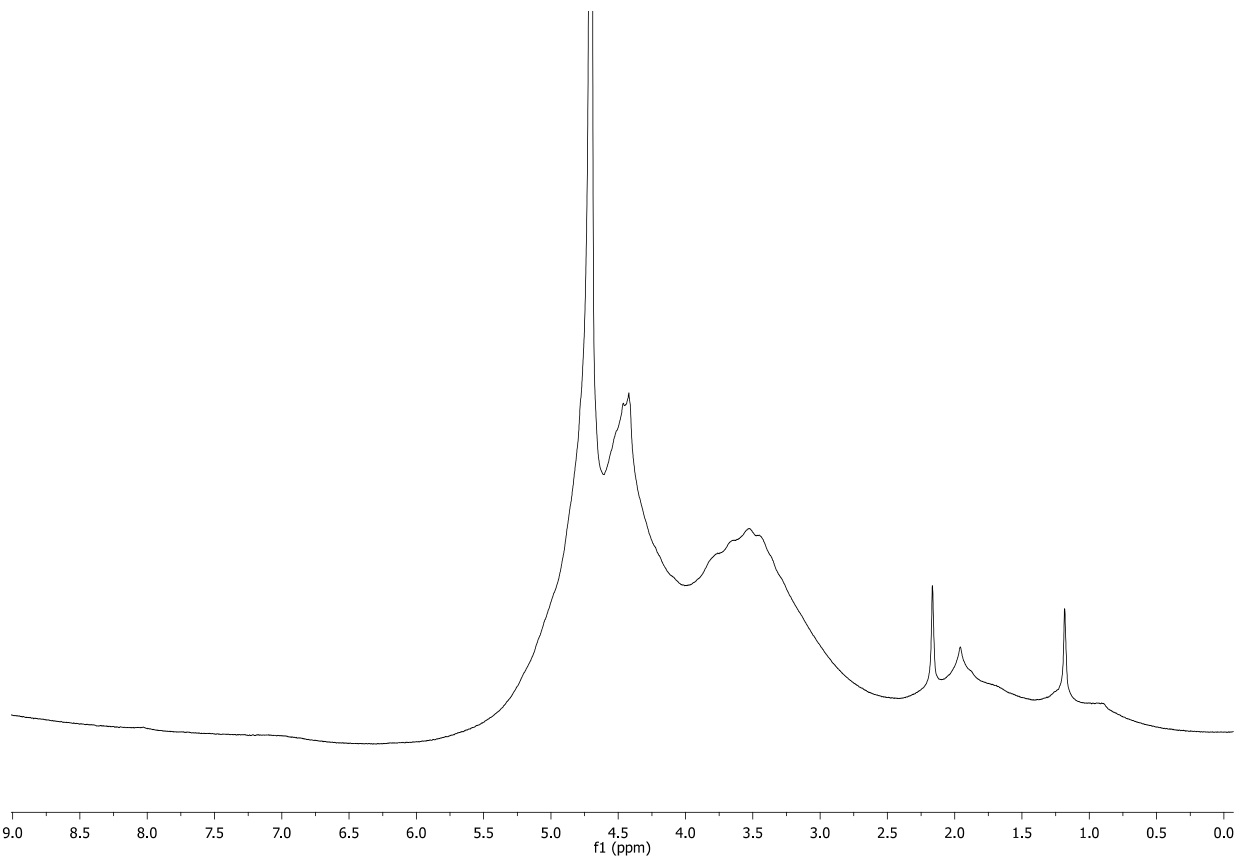

Figure S2. ^{1}H NMR (D_2O, 600 MHz) spectrum of the crosslinked material **HA(270)-FA-HEGEC-CL-10**.

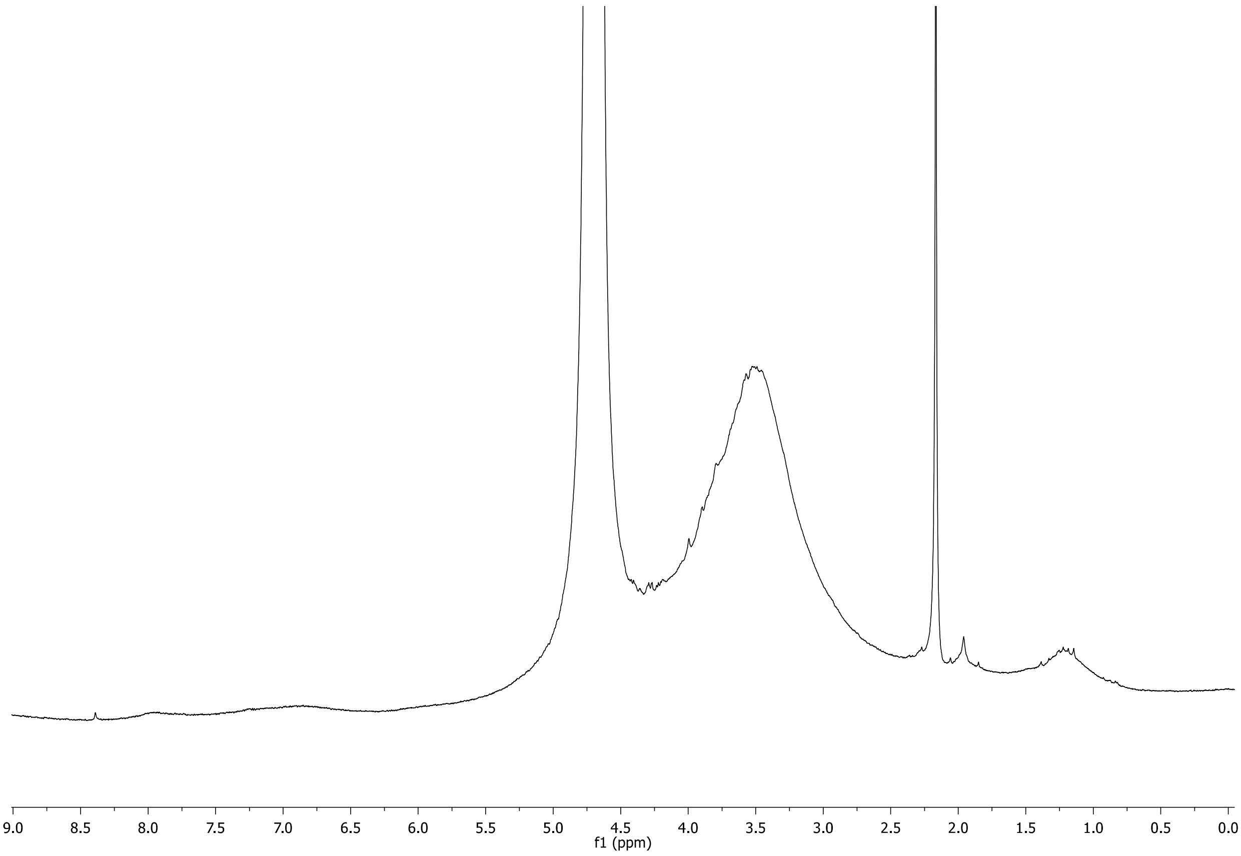

Figure S3. ^{1}H NMR (D_2O, 600 MHz) spectrum of the crosslinked material **HA(270)-FA-HEGEC-CL-40**.

Table S1. Z-average, PDI, and zeta-potential, of all the crosslinked materials produced (**HA(270)-FA-TEGEC-CL-10**, **HA(270)-FA-TEGEC-CL-20**, **HA(270)-FA-TEGEC-CL-40**, **HA(270)-FA-HEGEC-CL-10**, **HA(270)-FA-HEGEC-CL-20**, and **HA(270)-FA-HEGEC-CL-40**).

Sample	Z- average (nm)	PDI	Zeta potential (mV)
**HA(270)-FA-TEGEC-CL-10**	226.8 ± 15.8	0.312 ± 0.015	-41.4 ± 0.4
**HA(270)-FA-TEGEC-CL-20**	332.5 ± 22.6	0.338 ± 0.012	-39.1 ± 0.7
**HA(270)-FA-TEGEC-CL-40**	354.8 ± 25.8	0.456 ± 0.013	-35.8 ± 0.5
**HA(270)-FA-HEGEC-CL-10**	610.4 ± 19.5	0.240 ± 0.016	-41.6 ± 0.3
**HA(270)-FA-HEGEC-CL-20**	348.6 ± 18.4	0.279 ± 0.015	-41.5 ± 0.5
**HA(270)-FA-HEGEC-CL-40**	205.9 ± 28.7	0.247 ± 0.018	-36.5 ± 0.8
